# Amyloid oligomers as on-pathway precursors or off-pathway competitors of fibrils

**DOI:** 10.3389/fmolb.2023.1120416

**Published:** 2023-02-09

**Authors:** Martin Muschol, Wolfgang Hoyer

**Affiliations:** ^1^ Department of Physics, University of South Florida, Tampa, FL, United States; ^2^ Institut für Physikalische Biologie, Heinrich-Heine-Universität, Düsseldorf, Germany; ^3^ Institute of Biological Information Processing (IBI-7) and JuStruct, Jülich Center for Structural Biology, Forschungszentrum Jülich, Jülich, Germany

**Keywords:** amyloid, oligomers, energy landscape, protein aggregation, off-pathway oligomers

## Abstract

Amyloid Diseases involve the growth of disease specific proteins into amyloid fibrils and their deposition in protein plaques. Amyloid fibril formation is typically preceded by oligomeric intermediates. Despite significant efforts, the specific role fibrils or oligomers play in the etiology of any given amyloid disease remains controversial. In neurodegenerative disease, though, amyloid oligomers are widely considered critical contributors to disease symptoms. Aside from oligomers as inevitable on-pathway precursors of fibril formation, there is significant evidence for off-pathway oligomer formation competing with fibril growth. The distinct mechanisms and pathways of oligomer formation directly affect our understanding under which conditions oligomers emerge *in vivo,* and whether their formation is directly coupled to, or distinct from, amyloid fibril formation. In this review, we will discuss the basic energy landscapes underlying the formation of on-pathway *vs.* off-pathway oligomers, their relation to the related amyloid aggregation kinetics, and their resulting implications for disease etiology. We will review evidence on how differences in the local environment of amyloid assembly can dramatically shift the relative preponderance of oligomers *vs.* fibrils. Finally, we will comment on gaps in our knowledge of oligomer assembly, of their structure, and on how to assess their relevance to disease etiology.

## Introduction

Amyloid diseases involve a variety of structurally and functionally distinct proteins (Chiti and Dobson, 2017; Iadanza et al., 2018) which assemble into rigid, unbranching cross-β sheet fibrils and deposit as prominent proteinaceous plaques in the disease-affected tissues (Sunde et al., 1997; Lührs et al., 2005; Sawaya et al., 2007; Paravastu et al., 2008; Eisenberg and Jucker, 2012; Iadanza et al., 2018). The disruption of tissues due to the accumulation of fibril plaques was long considered the main pathogenic event. Over the past 20 years, though, accumulating evidence particular from neurodegenerative diseases and type-II diabetes has implicated small amyloid oligomers, instead of the prominent fibril deposits, as dominant pathogen (Dahlgren et al., 2002; Haass and Selkoe, 2007; Koffie et al., 2009; Kayed et al., 2010; He et al., 2012; Upadhaya et al., 2012; Kalia et al., 2013; Abedini et al., 2016; Koss et al., 2016; Sengupta et al., 2017; Cline et al., 2018; Rodriguez Camargo et al., 2018; Uhlmann et al., 2020; Schützmann et al., 2021; Emin et al., 2022). This “oligomer hypothesis” (Cline et al., 2018) provides one possible resolution to the known lack of correlation between post-mortem plaque load and neurological deficits in Alzheimer’s Disease (Braak and Braak, 1991). It received a significant boost by FDA approval of the antibody-drug lecanemab against Alzheimer’s Disease, which preferentially targets Aβ oligomers over monomers and fibrils (Swanson et al., 2021; Söderberg et al., 2022). Yet, this hypothesis has remained controversial (Benilova et al., 2012; Makin, 2018). This is, in part, due to the multitude of conceptual and practical challenges inherent to studying amyloid oligomers.

Here we will lay out some basic characteristics of amyloid oligomers. These, in turn, highlight some of the difficulties of defining what constitutes an amyloid oligomer, and why they are challenging to investigate, *in vitro* or *in vivo,* or to determine which ones among them are the most disease-relevant. We will discuss the distinct origins of oligomers as either on-pathway precursors or off-pathway competitors of fibril growth. We will consider three distinct energy landscapes underlying the three main models for on- or off-pathway oligomer formation. These different pathways of oligomer formation, in turn, imply differences of when and where amyloid oligomers arise *in vivo* and suggest distinct approaches required for targeting those oligomers most relevant to disease etiology. We will conclude by highlighting some gaps in our knowledge about oligomers and the experimental challenges in ascertaining the perhaps distinct roles amyloid oligomers play in different amyloid diseases.

## What defines amyloid oligomers?

One of the basic problems for investigating oligomers is the lack of universally agreed-upon criteria of what constitutes an amyloid oligomer. Here we propose three broad criteria encompassing a wide range of reported oligomer types (Harper et al., 1999; Stine et al., 2003; Gosal et al., 2005; Hill et al., 2009; Lorenzen et al., 2014). In general, amyloid oligomers.

• Are comparatively small, globular protein aggregates, or assemblies thereof.
• Are early-stage, metastable transients observed under amyloid growth conditions *in vitro*
• Have morphologies and structures distinct from fibrils.

The first criterion includes either multimolecular assemblies inherently limited in size due to specific (n-mers) or non-specific (micellar) binding. We do also include “beaded” curvilinear fibrils (often designated “protofibrils”) believed to be composed of strings of oligomers (Kodali and Wetzel, 2007; Hill et al., 2009; Schützmann et al., 2021). We exclude, however, droplet formation *via* liquid-liquid phase separation, as reported for tau (Ambadipudi et al., 2017; Wegmann et al., 2018; Kanaan et al., 2020), IAPP (Brender et al., 2015), FUS (Patel et al., 2015), and α-synuclein (Ray et al., 2020). While clearly relevant, liquid-liquid phase separation represents a distinct aggregation mechanism resulting in liquid droplets forming extended homogeneous phases (Kodali and Wetzel, 2007; Hill et al., 2009; Schützmann et al., 2021). Metastability indicates that oligomers either accumulate in front of an energy (nucleation) barrier and/or occupy a local free energy minimum distinct from the global minimum of fibril. We avoided the term “precursors” to indicate that oligomers can emerge as true on-pathway precursors or as off-pathway competitors of fibril formation. Here, “on-pathway” refers to oligomer populations with a finite probability for direct growth/conversion into the fibril state. “Off-pathway” classifies oligomers with zero probability for direct conversion into fibrils, i.e. without first completely dissociating into monomers. Consequently, the latter will intrinsically retard fibril formation by depleting the monomer pool for fibril nucleation and growth. Finally, we do not specify any structural characteristic for oligomers other than that they are distinct from fibrils. We sidestep any *a priori* distinction between toxic and non-toxic oligomers. This allows for the possibility that, dependent on the protein, its solution environment and its cellular co-factors, distinct oligomer species with distinct biological activities might emerge. It is the small (few nm) size and inherent metastability against fibril formation that has made detecting oligomers *in vitro* and *in vivo* challenging. As a result, there are only limited examples of high-resolution amyloid oligomer structures, yielding a diverse range of ordered and disordered structures (Laganowsky et al., 2012; Stroud et al., 2012; Apostol et al., 2013; Kotler et al., 2015; Fusco et al., 2017). Similarly, lower-resolution data of intermediates have found various structures, including anti-parallel beta-sheets (Cerf et al., 2009; Chandra et al., 2017; Hasecke et al., 2018), alpha-helices (Kirkitadze et al., 2001; Serra-Batiste et al., 2016) or disordered and micellar structures (Yong et al., 2002; Brender et al., 2015; Fusco et al., 2017; Morel et al., 2018).

In this review, though, we will discuss the distinct mechanisms proposed for early-stage oligomer formation either on-pathway or off-pathway from fibril formation, the corresponding free energy landscapes and how these properties affect their *in vitro* assembly kinetics and relative prevalence during fibril assembly. We will also discuss why these distinct mechanisms of oligomer formation inform potential approaches at targeting them *via* pharmacological interventions.

## On-pathway vs. off-pathway oligomer formation and the free energy landscapes of amyloid assembly

There are three main models that have been proposed for the formation of early-stage oligomers during *in vitro* fibril growth. These are nucleated polymerization, nucleated conformational conversion and off-pathway oligomer formation. Of these, the first two are on-pathway models in which oligomers are necessary precursors of fibril formation while the last one considers oligomers as distinct aggregate species incapable of converting/growing into fibrils. It is important to state upfront that on-pathway oligomers have to exist as true precursors to fibril formation. In addition, oligomer formation by any specific protein might well involve a mixture of these different pathways. The question is which model best represents the oligomer species experimentally observed during *in-vitro* experiments and, furthermore, which ones among them produces the disease-relevant species?

**FIGURE 1:**
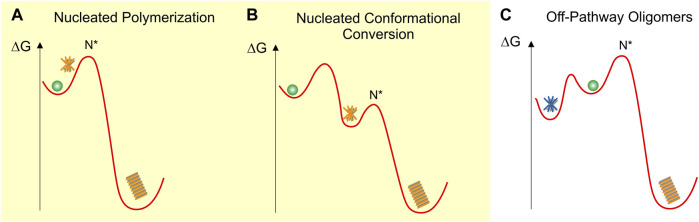
Schematic fee-energy landscapes for oligomer formation **(A)** On-pathway Nucleated Polymerization or NP, **(B)** On-pathway Nucleated Conformational Conversion or NCC **(C)** Off-pathway oligomer formation. Oligomers are indicated as orange (on-pathway) or blue (off-pathway) stars and fibrils as orange stacks. N* indicates the critical fibril nucleus at the peak of the energy barrier ΔG_f_ for fibril formation. The energy barriers between the different states are not to scale and are strongly dependent on solution conditions. The various oligomer and fibril energy minima themselves are likely to be “rugged,” allowing for multiple energetically similar oligomer and fibril polymorphs.

We will discuss these different models in the context of their underlying schematic free energy landscapes (Figure 1). In all cases, fibril growth involves a (primary) nucleation process with a free energy barrier (ΔG_1st_). While not included in these schematics, the highly sigmoidal kinetics of fibril nucleation further imply the presence of fibril-mediated secondary nucleation mechanisms (ΔG_2nd_) that lower the nucleation barrier for new seed formation (Ferrone et al., 1985; Knowles et al., 2009).

### Nucleated polymerization (NP)

In nucleated polymerization (Figure 1A), monomers are separated from fibrils by a nucleation barrier. In this scenario, amyloid oligomers are pre-nucleation clusters forming in front of the nucleation barrier. These pre-nucleation cluster, prior to reaching the size of the critical nucleus N*, are likely to be structurally diverse and distinct from fibrils. The critical nucleus N*, by definition, has a 50-50 chance of either decaying back to the monomer state or growing into a mature fibril. Similar to classical nucleation theory (Sear, 2007) both the populations and lifetimes of such pre-nucleation clusters are severely constrained by the unfavorable Boltzmann factor related to the energy barrier in front of N*. Therefore, these transient pre-nucleation oligomers are inherently difficult to detect experimentally. This seems distinct from the long-lived oligomers observed both *in vitro* (Dahlgren et al., 2002; Stine et al., 2003; Hill et al., 2009; Perez et al., 2019) and *in vivo* (Dettmer et al., 2013; Esparza et al., 2016; Röhr et al., 2020). Small post-nucleation aggregates emerging on the other side of N* in this model are transient and might have biological activities distinct from their mature counterparts (Xue et al., 2009). However, they already share the structure and morphology of mature fibrils and are not metastable. They therefore do not match the definition for “oligomers.” If they *are* metastable and structurally and morphologically distinct from fibrils, they will be subsumed under the nucleated conformational conversion model discussed next.

### Nucleated conformational conversion (NCC)

On-pathway NCC was proposed originally to explain the significant populations of well-defined and fairly long-lived globular oligomers observed *in vitro* with the yeast protein sup35 (Serio et al., 2000). This model has also been applied to explain experimental data on oligomer formation by Aβ40 and Aβ42 using a combination of fluorescence kinetics and high-resolution imaging approaches (Ahmed et al., 2010; Lee et al., 2011; Fu et al., 2015; Lee and Terentjev, 2017; Nick et al., 2018). The free energy landscape for the model of nucleated conformational conversion (NCC) is shown in Figure 1B (Serio et al., 2000; Lee et al., 2011; Fu et al., 2015). In contrast to the NP landscape, oligomers form in a local free energy minimum along the fibril assembly path. This energy minimum allows oligomers to accumulate in significant numbers and form long-lived, metastable populations. The fibril nucleation barrier now represents the structural conversion of these on-pathway oligomers from an oligomer-specific structure and morphology to the cross-β sheet structure of the mature fibrils.

### Off-pathway oligomers (off-Os) and nucleated polymerization (NP)

Figure 1C represents the free-energy schematic for nucleated polymerization in which oligomers accumulate in a local free energy minimum off-pathway from fibril nucleation (Powers and Powers, 2008). At first glance, NCC and off-O models might appear only superficially different. Similar to surfactant micelles, the globular morphology and limited size of these oligomers is likely the direct result of their size-dependent free energy minimization. Even with these limitations their internal structures could be quite varied and allow for significant polymorphism. The observation that oligomer and fibril formation proceed under comparable environmental conditions implies that, just as fibrils, off-Os require a non-native conformation and sufficient flexibility of the amyloid protein to assume the oligomer structure. Experimentally, it is challenging to distinguish NCC and off-Os from one another. Both models allow for the accumulation of significant populations of oligomers in a local free energy minimum and they predict similar progressions in observable oligomer *vs.* fibril populations.

### On-pathway vs. off-pathway oligomers as therapeutic targets

Despite the above similarities, there are important differences between on- and off-pathway oligomers. On-pathway oligomers, by definition, are prerequisite precursors of fibril formation. Therefore, increases in on-pathway oligomers should increase the probability for net conversion into fibrils and, thereby, accelerate fibril growth. In contrast, off-Os are inherently inhibitory to fibril formation since they compete for and deplete the pool of monomers available for fibril nucleation and elongation. Even after fibril nucleation, the metastability and off-pathway characteristics of off-Os collude to increase their inherent lifetimes. Once fibrils have depleted the available pool of free monomers to grow from, the only way they can continue to grow is by monomers dissociating from existing off-Os. Yet, the metastability of oligomers makes this an inherently slow process.

As a result of the uncoupling of off-O and fibril formation, off-Os can emerge and thrive under conditions distinct from those favorable to fibril formation. In contrast, on-pathway oligomer formation and fibril population are strictly correlated. As a result, levels of off-Os present in patients can be uncorrelated with their AD plaque burden seen *post mortem*. Whether disease-relevant amyloid oligomers are on-pathway or off-pathway from fibril formation also has fundamental implications on how to target them with therapeutic interventions. For on-pathway oligomers, interventions targeting oligomer formation directly are bound to suppress fibril formation at the same time. Suppression of on-pathway oligomers and fibrils, in turn, would increase the pool of free monomers available for off-pathway oligomers to emerge. Conversely, selectively targeting off-Os would tend to enhance on-pathway oligomers and fibril formation. In addition, a decrease of amyloid plaque loads, as observed in PET scans, could be indicative of a decrease or increase in oligomer population.

### Experimental data on amyloid kinetics and mechanisms of oligomer formation

Pre-nucleation oligomers accumulating in front of the nucleation barrier, as indicated by NP, have to exist. However, just like pre-nucleation clusters during crystallization (Gebauer et al., 2014), they are notoriously difficult to detect experimentally. They are expected to have very short lifetimes, have high turnover (association and dissociation rates), and their populations are inherently limited by the unfavorable Boltzmann factor of the free energy barrier. In kinetic experiments, ThT does not have sufficient sensitivity for (or might not even bind to) pre-nucleation clusters during the lag-phase of fibril formation. In addition, one would have to be able to discriminate signals from pre-nucleation clusters from fibrils formed *via* primary nucleation during the lag phase. For the above reasons, it seems likely that amyloid oligomers observed in many *in vitro* kinetic experiments are formed in a local free energy minimum, such as presumed by NCC or off-pathway oligomer formation. There are some kinetic experiments, though, that have used alternative approaches to monitor the kinetics of pre-nucleation oligomers, and their subsequent decay following fibril nucleation and growth (Karamanos et al., 2019).

**FIGURE 2:**
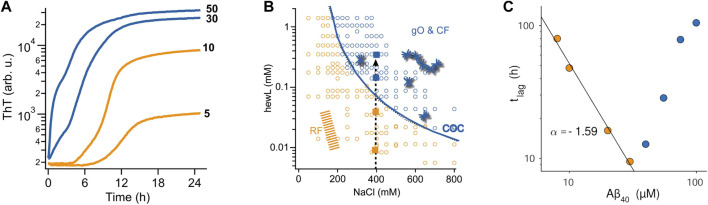
Effects of oligomers on the kinetics of fibril assembly **(A)** Kinetic transition from purely sigmoidal (orange) to biphasic (blue) ThT kinetics for Aβ42 assembly at pH 7.4 (50 mM sodium phosphate) and 27°C. ThT fluorescence is plotted logarithmically to highlight the flatness during the lag phase in the sigmoidal regime. As discussed, this kinetic transition is consistent with both NCC and off-O formation **(B)** Kinetic “phase diagram” for the onset of biphasic oligomer formation (blue line) during lysozyme fibril growth at pH 2. While fibrils (orange & blue dots) will eventually form for all protein/salt combinations in this diagram, biphasic oligomers only emerge beyond the conditions outlined by the solid blue line. This uncoupling of favorable conditions for fibril vs. oligomer is another indicator for off-pathway oligomers. **(C)** Plot of the lag phase for fibril formation of Aβ40 at pH 7.4 as function of protein concentration. In the sigmoidal regime (orange dots) the lag phase decreases as a power-law in protein concentration (black line), as expected for nucleated polymerization. The onset of biphasic oligomer formation (blue dots) reverses this trend. This inhibitory effect on fibril nucleation is a strong indication for off-pathway oligomers. Figures 2A, C are adapted from ref (Hasecke et al., 2021) and Figure 2B is adapted from (Hasecke et al., 2018).

Using atomic force microcopy Lee et al. described noticeable globular oligomer populations in the ThT lag-phase of Aβ40 assembly. Using a FRET probe they were able to detect the kinetics of these “lag-phase oligomers” (Lee et al., 2011). When used as seeds, these lag-phase aggregate populations did accelerate fibril formation, suggesting that they were on-pathway. At the same time, there are good indications from multiple amyloid proteins that the metastable oligomers envisioned in NCC and off-O NP cause a build-up in oligomer populations significant-enough to cause a transition in ThT-monitored amyloid assembly from sigmoidal to biphasic kinetics (Figure 2A) and to be picked up even by light scattering (Hill et al., 2011; Lee et al., 2011; Foley et al., 2013). The changes in ThT kinetics range from subtle lag-free ThT drifts to rapid upswings and intermediate plateaus (Hortschansky et al., 2005; Cloe et al., 2011; Fu et al., 2015; Miti et al., 2015; Hasecke et al., 2018; Nick et al., 2018; Chen et al., 2019; de Oliveira and Silva, 2019; Shea et al., 2019; Hasecke et al., 2021; Schützmann et al., 2021). Such biphasic ThT kinetics emerged at elevated concentrations in both Aβ40 and Aβ42, and was interpreted as support for the NCC model of fibril formation (Fu et al., 2015; Nick et al., 2018). For lysozyme and Aβ40, the concentration-dependence of biphasic oligomer kinetics was shown to follow n^th^ order reaction kinetics (Miti et al., 2015; Hasecke et al., 2018). This indicates the cooperative and micelle-like characteristics of “biphasic” oligomer formation. By themselves, though, the presence of biphasic kinetics, the observed temporal progression from oligomers to fibrils, or associated changes in structural characteristics derived from spectroscopic methods don't allow discriminating between on- or off-pathway oligomers.

As indicated above, solution conditions permissive of fibril and off-O formation do not have to coincide. For biphasic oligomers of lysozyme and Aβ it was shown indeed that there was a threshold protein concentration, called “critical oligomer concentration” or COC, for the onset of oligomer formation. This COC was clearly distinct from the onset of fibril formation (Hasecke et al., 2018; Hasecke et al., 2021) and highly sensitive to changes in the solution environment (Figure 2B) (Miti et al., 2015; Hasecke et al., 2021). For β2-microglobulin, Gosal et al. mapped out a pH-dependent kinetics phase diagram of distinct aggregate morphologies emerging at different regions of a three-parameter pH-salt-protein phase space. They categorized them as “worm-like,” “rod-like” and “long-straight” aggregates(Gosal et al., 2005). Among those, only worm-like and rod-like aggregates reacted with an anti-oligomer antibody. They also showed that neither of these two types were precursors (“protofibrils”) of fibril formation.

The other indicator for off-Os is their anticipated concentration-dependent inhibition of fibril nucleation and growth. The intrinsic inhibitory nature of off-Os are the result of their depletion of the monomer pool available for fibril nucleation and growth (Powers and Powers, 2008; Perez et al., 2019), their inherent metastability which retards their fibril-induced dissociation back to monomers, and their reported ability to bind to fibrils and suppress secondary nucleation events (Hasecke et al., 2018; Hasecke et al., 2021). Intriguingly, the original paper on NCC oligomers already noted that increased oligomer populations of sup35 caused a delay instead of an acceleration in fibril formation (Serio et al., 2000). Similar evidence for off-pathway oligomer formation has been provided for a variety of amyloid proteins (Souillac et al., 2002; Gosal et al., 2005; Jahn and Radford, 2008; Hill et al., 2011; Foley et al., 2013; Miti et al., 2015; Hasecke et al., 2018; Hasecke et al., 2021). Souillac et al. noted that the lag period for fibril formation by immunoglobulin light chain was dramatically increased with increasing monomer concentration (Souillac et al., 2002). Similarly, the onset of biphasic kinetics for lysozyme amyloid assembly at pH 2 as well as for Aβ42, Aβ40, and a dimeric variant of Aβ40 all resulted in a significant increase in the lag period for fibril formation with increasing protein concentration (Figure 2C).

## Conclusion and outlook

The above discussion of distinct pathways for *in vitro* amyloid oligomer formation highlighted why their differences are likely to translate into distinct approaches towards targeting amyloid oligomers pharmacologically. The strong dependence of the protein threshold for oligomer formation on localized amyloid concentrations and solution conditions, in particular pH, match with direct observation that conditions for preferential oligomer formation *in vivo* involve acidic cellular compartments (Hu et al., 2009; Schützmann et al., 2021). Similarly, changes in ionic composition, pH and Aβ expression can occur transiently in the extracellular space during common risk factors of AD, including ischemic stroke, traumatic brain injury, and migraine (Blasko et al., 2004; Pluta et al., 2013; Morton et al., 2019). Therefore, efforts at unraveling the mechanisms of amyloid oligomer formation *in vitro* are important for elucidating the complex mechanisms of oligomer formation *in vivo*.

There remain a lot of open questions, though, relating to the role of oligomer formation in disease. For one, confirming the pathogenic relevance of oligomers will likely require the development of assays and probes for detecting oligomers *in vivo*. Some fluorescent indicators have shown promise at discriminating distinct amyloid aggregate populations (Rasmussen et al., 2017; Barton et al., 2019). This raises the additional complications of oligomer polymorphism. On-vs. off-pathway oligomers are unlikely to share a common structure. Even within these two categories there are indications for distinct oligomer polymorphs (Fusco et al., 2017; König et al., 2021; Niyangoda et al., 2021). Beyond the conformation-specific antibodies (Kayed and Glabe, 2006), recent advances in spectroscopic approaches *in vitro* (Ruggeri et al., 2015) and *post mortem* (Röhr et al., 2020) are likely to provide novel insights into structural polymorphism and its relevance to oligomer formation *in vivo*. In the end, progress on all these fronts will be required to identify which oligomer categories are most disease-relevant, and in which amyloid diseases.
